# Whole genome sequencing of penicillin-resistant *Streptococcus pneumoniae *reveals mutations in penicillin-binding proteins and in a putative iron permease

**DOI:** 10.1186/gb-2011-12-11-r115

**Published:** 2011-11-22

**Authors:** Fereshteh Fani, Philippe Leprohon, Danielle Légaré, Marc Ouellette

**Affiliations:** 1 Centre de recherche en Infectiologie du Centre de recherche du CHUL and Département de Microbiologie, Infectiologie et Immunologie, Faculté de Médecine, Université Laval, 2705 Boul. Laurier, Québec, Canada

## Abstract

### Background

Penicillin resistance in *Streptococcus pneumoniae *is mediated by a mosaic of genes encoding altered penicillin-binding proteins (PBPs). Nonetheless, *S. pneumoniae *has also developed non-PBP mechanisms implicated in penicillin resistance. In this study, whole genome sequencing of resistant organisms was used to discover mutations implicated in resistance to penicillin.

### Results

We sequenced two *S. pneumoniae *isolates selected for resistance to penicillin *in vitro*. The analysis of the genome assemblies revealed that six genes were mutated in both mutants. These included three *pbp *genes, and three non-*pbp *genes, including a putative iron permease, spr1178. The nonsense mutation in spr1178 always occurred in the first step of the selection process. Although the mutants had increased resistance to penicillin, the introduction of altered versions of PBPs into a penicillin-susceptible strain by sequential transformation led to strains with a minimal increase in resistance, thus implicating other genes in resistance. The introduction by transformation of the non-PBP recurrent mutations did not increase penicillin resistance, but the introduction of the nonsense mutation in the putative iron permease spr1178 led to a reduced accumulation of reactive oxygen species following exposure to penicillin and to other bactericidal antibiotics as well.

### Conclusions

This study indicates that the selection of resistance to penicillin in *S. pneumoniae *involves the acquisition of mutations conferring tolerance to the antibiotic-induced accumulation of oxidants, which translates into an increased survival that putatively enables the selection of major resistance determinants such as mutations in PBPs.

## Background

*Streptococcus pneumoniae *is an important pathogen of the respiratory tract causing community-acquired pneumonia worldwide [1]. It is also an etiological agent of otitis media, sepsis, and meningitis in adults and children and constitutes a significant health threat. Penicillin, a ß-lactam antibiotic, has long been the mainstay against pneumococcal infections but its efficacy is threatened by the rapid dissemination of penicillin non-susceptible clones worldwide, the prevalence of which varies between countries (reviewed in [2,3]).

β-Lactams are bactericidal antibiotics that inhibit the synthesis of the peptidoglycan layer of bacterial cell walls by inactivating penicillin-binding proteins (PBPs), a group of membrane-associated cytoplasmic proteins involved in the assembly of peptidoglycan and whose inhibition results in growth arrest and lysis. Resistance to ß-lactam antibiotics in clinical isolates of *S. pneumoniae *occurs through the acquisition of mosaic genes encoding altered PBPs. The mosaic genes encode PBP variants of lower antibiotic binding affinities and are the result of intra- and interspecies gene transfer events involving related streptococcal species [4,5]. Although *S. pneumoniae *contains six PBPs, variants of PBP2x, PBP2b and PBP1a are considered the most relevant to penicillin resistance. Furthermore, the acquisition of low-affinity PBP2x and PBP2b variants was shown to be a prerequisite for PBP1a variants to confer high-level resistance to ß-lactams [6,7]. PBP2x has the highest affinity for penicillin in *S. pneumoniae *and a variety of amino acid substitutions interfering with the polarity and charge distribution in the vicinity of the active site have been implicated in poor antibiotic binding and resistance [8,9].

Mutations in PBPs are not the sole contributors of resistance to ß-lactams in *S. pneumoniae*, and evidence for some non-PBP resistance mechanisms is available. Indeed, the cell wall of penicillin non-susceptible isolates is often highly enriched in branched chain muropeptides, a phenomenon linked to mosaic alleles of the *murM *gene [10,11]. Furthermore, mutations in a peptidoglycan N-acetylglucosamine deacetylase [12], a peptidoglycan O-acetyltransferase [13], a putative glycosyltransferase [14], a serine threonine kinase [15], a histidine protein kinase part of a two-component signal transducing system [16], or in a phosphate ABC transporter [17] have been implicated in resistance to ß-lactams.

Global approaches such as whole genome sequencing (WGS) of antibiotic-sensitive and -resistant isolates are powerful tools that are now readily available for use in determining the mode of action of antimicrobial drugs and the mechanisms involved in resistance [18-20]. We report here the WGS of two independent *S. pneumoniae *mutants selected for *in vitro *resistance to penicillin and the identification of known and new mutations involved in resistance.

## Results

### Selection and whole-genome sequencing of *S. pneumoniae *penicillin non-susceptible mutants

Two independent penicillin-resistant mutants of *S. pneumoniae *R6 and *S. pneumoniae *1974 were selected by stepwise penicillin increments until they reached a final penicillin minimum inhibitory concentration (MIC) of 2 μg/ml. It has not been possible to obtain mutants resistant to higher levels. The penicillin MICs of the wild-type (WT) progenitors were 0.023 μg/ml and 0.016 μg/ml for the *S. pneumoniae *R6 and 1974 lineages, respectively. The most highly resistant isolates were named R6M1 and R6M2 or 1974M1 and 1974M2 depending on whether they were derived from the *S. pneumoniae *R6 or 1974 background, respectively. All four penicillin-resistant strains were cross resistant to the cefotaxime but remained susceptible to erythromycin, tetracycline, linezolid, kanamycin and ciprofloxacin (Table 1).

**Table 1:** Susceptibility levels of *S.pneumoniae *isolates

*S. pneumoniae*	MIC (μg/ml)						
strain	PG	CT	EM	CI	KM	TC	LZ
R6-WT	0.023	0.023	0.125	0.5	25	0.125	0.38
R6M1	2.0	0.75	0.125	0.5	25	0.125	0.38
R6M2	2.0	0.75	0.125	0.5	25	0.125	0.38
1974-WT	0.016	0.023	0.125	0.5	25	0.125	0.75
1974M1	2.0	1.5	0.125	0.5	25	0.125	0.75
1974M2	2.0	2.0	0.125	0.5	25	0.125	0.75

Results are the average of at least three independent measurements. CI, ciprofloxacin; CT, cefotaxime; EM, erythromycin; KM, kanamycin; LZ, linezolid; PG, penicillin G; TC, tetracycline; WT, wild-type.

We conducted WGS of R6M1 and R6M2 in order to elucidate the genetic events associated with the penicillin-resistant phenotype. The genome of R6M1 was sequenced by using the comparative genome sequencing technology developed by NimbleGen, which relies on the use of tiled DNA microarray hybridizations to rapidly survey entire microbial genomes and to identify the location of SNPs, insertions, or deletions [19,20]. The comparative genome sequencing of R6M1 and WT parent allowed the identification of 26 mutations in R6M1 (Table 2) that were further confirmed by PCR amplification and conventional DNA sequencing. The genome of R6M2 was sequenced using the massively parallel 454 Life Science (Roche) GS-FLX DNA sequencing platform, which generated a genome assembly of 28× coverage, with 97% of the reads assembled into 78 large contigs. Comparative sequence analysis of R6M2 and its R6 WT parent revealed 52 mutations (Table 2) that were confirmed by PCR amplification and conventional DNA sequencing. The mutations can also be seen as part of circular schematic maps (Additional file 1).

**Table 2:** Mutations identified in R6M1 and R6M2 penicillin-resistant mutants

		*S. pneumoniae *strain			
		M1		M2	
**Locus name**	**Putative identification**	**Nucleotide change**	**Amino acid change**	**Nucleotide change**	**Amino acid change**
spr0032	DNA polymerase I			A107G	H36R
spr0041	Transposase (orf2)			GAA31AAC	E11N
				GCTCG36TCTCA	K12N;D14N
				T63A	SYN
				GG204TA	D69Y
spr0113	Hypothetical protein			A4G	K2E
spr0121	Surface protein PspA precursor			A4G	N2D
				T1374C	SYN
spr0160	DNA mismatch repair protein (Hex B)			G752A	S251N
spr0182	Hypothetical protein			G82insertion	frame shift
spr0284	Alpha-xylosidase			T1107C	SYN
spr0304	Penicillin-binding protein 2x	A1150G	R384G	A842C	Q281P
		G1552T	V518L	C1106T	A369V
		C1654G	Q552E	A1150G	R384G
				C1276T	R426C
				G1552A	V518I
spr0329	Penicillin-binding protein 1a	G1630A	G544R	G1233A	W411*
spr0376	Conserved hypothetical protein			T207C	SYN
spr0422	Hypothetical protein			C391T	Q131*
spr0475	Conserved hypothetical protein	G318C	L106F		
spr0509	Phenylalanyl-tRNA synthetase beta chain			C105T	SYN
spr0598	GTP-binding protein (TypA/BipA) (tyrosine phosphorylated protein A)			C905T	P302L
spr0666	ABC transporter ATP-binding protein - cell division (FtsE)	C410T	P137L		
spr0764	30S Ribosomal protein S1			G1173A	M391I
spr0776	D-Alanyl-D-alanine carboxypeptidase			C287T	A96V
spr0866	Dihydroorotate dehydrogenase	C575T	P192L		
spr0878	Exoribonuclease R	G280T	G94W		
spr0895	Conserved hypothetical protein			G134C	R45T
spr0917	Citrulline cluster-linked gene	C59T	A20V		
spr0934	ABC transporter substrate-binding protein - iron transport			G486A	W162*
spr1041	Hypothetical protein	C648T	SYN		
spr1043	Conserved hypothetical protein	C109T	P37S	C105T	SYN
spr1092	tRNA pseudouridine 5S synthase			C210T	SYN
spr1127	Ribonuclease III	A228G	SYN	C554T	T185I
spr1152	LicD	G692T	P231L		
spr1166	Signal recognition particle (Fifty four homolog)	C139T	P47S		
spr1178	Hypothetical protein	C82T	Q28*	C82T	Q28*
spr1186	N-Acetylneuraminate lyase subunit, truncation			T603C	SYN
				G620A	G207D
				GAC660AAT	T221I
				AA689CG	E230A
				T696A	SYN
spr1224	Conserved hypothetical protein			T564C	SYN
spr1240	Alanyl-tRNA synthetase			A139G	T47A
spr1254	ABC transporter ATP-binding protein-phosphate transport (PstB)	G499A	G167S	C614T	T205I
spr1260	Conserved hypothetical protein			T597C	SYN
spr1272	N-Acetylglucosamine-6-phosphate isomerase	G385A	G129R		
spr1384	UDP-N-acetylmuramoyl-L-alanyl-D-glutamyl-L-lysine ligase	G996A	M332I		
spr1423	Conserved hypothetical protein			A673G	M225V
spr1453	Major facilitator superfamily transporter	T91C	F31L		
spr1465	Conserved hypothetical protein	G419A	G140E		
spr1517	Penicillin-binding protein 2b	C1245A	D415E	C1184T	A395V
		C1528A	G665D	G1303A	G435S
				A1351G	T451A
spr1587	Conserved hypothetical protein	C568T	SYN		
spr1703	ABC transporter ATP-binding protein - oligopeptide transport	C575A	P192Q		
spr1706	ABC transporter membrane-spanning permease - oligopeptide transport			T399deletion	frameshift
spr1862	Competence protein			A51G	SYN
spr1886	Degenerate transposase	A314G	*105W		
spr1888	DNA mismatch repair protein (HexA)	C2183T	T728I	C976T	Q326*
spr1991	Glycerol kinase			GG78TT	E27*

Asterisks and SYN indicate nonsense mutations and synonymous mutations, respectively.

The WGS of R6M1 and R6M2 identified a total of 40 genes that have acquired a non-synonymous mutation in at least one of the mutants (Table 2). Of these, six genes were mutated in both mutants (Table 3). These included three PBP-encoding genes, *pbp2x*, *pbp2b *and *pbp1a*, in which a total of 14 missense mutations and one nonsense mutation have been observed in R6M1 and R6M2 (Table 3). The targeted sequencing of the six common genes in the 1974M1 and 1974M2 strains identified another 14 missense substitutions in PBP2x and PBP2b and nonsense mutations in PBP1a (Table 3). The T451A and G435S amino acid substitutions in PBP2b and the R384G, V518I and Q552E substitutions in PBP2x were shared by some of the mutants derived from R6 and 1974 (Table 3). The three other non-PBP-encoding genes identified by WGS mutated in both R6 mutants (although not always at the same position) were the ABC protein PstB (spr1254), the DNA mismatch repair protein HexA (spr1888) and a hypothetical protein (spr1178). Interestingly, the analysis of targeted PCR fragments from 1974M1 and 1974M2 revealed that the same nonsense mutation occurred at position 28 of the spr1178 protein in all mutants but no mutations were seen in spr1254 and spr1888 in the 1974 mutants (Table 3).

**Table 3:** Genes mutated in at least two *S.pneumoniae *penicillin resistant mutants

	*S. pneumoniae *strains			
Locus name	R6M1	R6M2	1974M1	1974M2
PBP1a	G544R	W411*	E248*	E158*
PBP2x	**R384G**,**V518I**, **Q552E**	Q281P, A369V, **R384G**, R426C, **V518I**	F388L, **Q552E**, V573L, V587L, G601V	A507V, P535L, **Q552E**
PBP2b	D415E, G665D	A395V, G435S, **T451A**	G435S, **T451A**	T431D, **T451A**, L492F, Q633E
Spr1178	Q28*	Q28*	Q28*	Q28*
Spr1254	G167S	T205I	WT	WT
Spr1888	T728I	Q326*	WT	WT

Mutations are shown as amino acid changes with their corresponding position in the protein. Asterisks indicate nonsense mutations. Mutations that are common in at least two strains are in bold.

### Reconstruction of resistance by transformation of mutated PBPs

Transformation experiments of *S. pneumoniae *R6 WT with *pbp *genes amplified from either the R6M1 or R6M2 mutants were conducted to assess the contribution of the different PBP mutations to penicillin resistance. The analysis of the PBP sequences at the different levels of R6M1 and R6M2 selection (0.06, 0.125, 0.25, 0.5, 1.0 and 2.0 μg/ml penicillin G (PG)) revealed a stepwise selection of PBP mutations (Additional file 2). The analysis of the chronological appearance of PBP mutations in R6M2 revealed that the progression towards penicillin resistance began with the Q281P substitution in PBP2x followed by the T451A substitution in PBP2b. The other PBP2x mutations happened sequentially as the level of resistance to penicillin increased and the remaining PBP2b and PBP1a mutations were only selected at high concentration of penicillin (Additional file 2). The *pbp2x*, *pbp2b *and *pbp1a *genes were amplified from R6M1 and R6M2 genomic DNA and were sequenced to confirm the presence of the mutations described in Table 3. Because of the order of mutation appearance (Additional file 2), we introduced sequentially the R6M1 mutations by first transforming the PCR fragment for *pbp2x *into the recipient *S. pneumoniae *R6 WT. The selection of transformants with 0.03 μg/ml penicillin enabled the transfer of the three PBP2x mutations found in R6M1 (R384G, V518L, Q552E). This transformant, named R6^2x-M1^, had a penicillin MIC of 0.06 μg/ml (Table 4). In a second round of transformation, the *pbp2b *gene from R6M1 was used as donor DNA for the transformation of the recipient R6^2x-M1^. Selection with 0.06 μg/ml penicillin yielded second-level transformants that acquired the two PBP2b mutations of R6M1 (D415E, G665D) and these transformants, called R6^2x2b-M1^, had a penicillin MIC of 0.125 μg/ml (Table 4). All attempts to introduce *pbp1a *mutations failed. We used a similar approach for R6M2 but in the first level transformation we pooled the *pbp2x*, *2b *and *1a *PCR fragments derived from R6M2 that were transformed into R6WT. The selection of transformants with 0.03 μg/ml penicillin enabled the transfer of only the PBP2x mutations Q281P, A369V and R384G, but not the R426C and V518I mutations despite several attempts. The transformants, named R6^2x-M2^, had a penicillin MIC of 0.06 μg/ml (Table 4). In a second round of transformation, a pool of *pbp2b *and *pbp1a *PCR fragments from R6M2 was used as donor DNA in the transformation of the recipient R6^2x-M2^. Selection with 0.06 μg/ml penicillin yielded second-level transformants that acquired the three PBP2b mutations of R6M2 (T451A, G435S and A395V) but retained an unaltered allele of *pbp1a*. These transformants, named R6^2x2b-M2^, had a penicillin MIC of 0.125 μg/ml (Table 4). Several attempts failed to introduce the PBP1a mutation of R6M2 into the R6^2x2b-M2 ^line.

**Table 4:** Minimal inhibitory concentrations to penicillin of *Streptococcus pneumoniae *mutants and transformants

Strain or transformant	Description	MIC to PG (μg/ml)^a^
R6	Wild type	0.023
CCRI1974	*S. pneumoniae*, sensitive clinical isolate	0.023
R6M1	R6 clone selected *in vitro *for PG resistance	2.0
R6M2	R6 clone selected *in vitro *for PG resistance	2.0
1974M1	1974 clone selected *in vitro *for PG resistance	2.0
1974M2	1974 clone selected *in vitro *for PG resistance	2.0
CP1250	*S. pneumoniae *that contains a spontaneous mutation in the *rpsL *gene that confers resistance to SM	
R6^2x-M1^	R6-WT transformed with *pbp2x *PCR fragments from R6M1 (contains all three missense mutations present in PBP2x of R6M1)	0.06
R6^2x-M2^	R6-WT transformed with *pbp2x *PCR fragments from R6M2 (contains just three mutations - Q281P, A369V, R384G - out of five mutations present in PBP2x of R6M2)	0.06
R6^2x2b-M1^	R6^2x-M1 ^transformed with *pbp2b *PCR fragments from R6M1 so all missense mutations present in PBP2b of R6M1 transformed into this transformant	0.125
R6^2x2b-M2^	R6^2x-M2 ^transformed with *pbp2b *PCR fragments from R6M2 so all missense mutations present in PBP2b of R6M2 transformed into this transformant	0.125
R6^2x2b-M2, 1a::Janus^	R6^2x2b-M2 ^knocked out in *pbp1a *by Janus cassette, KM^R^SM^S^	0.125
R6^SmR, 2x2b1a-M2,^	R6^2x2b-M2, 1a::Janus ^subjected to second step transformation with *pbp1a *of the Janus cassette with *pbp1a *of R6M2, KM^S^SM^R^	0.125
R6^SmR, 2x2b1a,spr1178-M2,^	R6^2x2b1a-M2 ^transformed with spr1178 PCR fragments from R6M2 (contains a Q28* nonsense mutation in spr1178), SM^R^	0.125
R6^2x2b,spr1254-M2^	R6^2x2b-M2 ^transformed with *pstB *PCR fragment from R6M2 (contains a T205I mutation in PstB)	0.125
R6^2x2b, spr1254, spr1178-M2^	R6^2x2b-M2 ^transformed with spr1178 and *pstB *PCR fragments from R6M2 (contains the substitutions Q28* in spr1178 and T205I in PstB)	0.125
R6M1^SmR,1a-WT^	R6M1 co-transformed with a *pbp1a *PCR fragment from R6-WT and a *rpsL *streptomycin resistance marker, SM^R^	1.0
R6^ko in spr1178^	R6-WT knocked out in spr1178, CM^R^	0.023
R6^ko in spr1254^	R6-WT knocked out in spr1254, KM^R^	0.023
R6^spr1888::janus^	R6-WT knocked out in spr1888 by Janus cassette, KM^R^SM^S^	0.023

^a^Results are the average of at least three independent measurements. Asterisks indicate nonsense mutations. PG, penicillin; KM, kanamycin; SM, streptomycin.

Given that PBP1a variants are usually associated with high level β-lactam resistance, we assessed the role of the R6M2 *pbp1a *point mutation by transforming the R6^2x2b-M2 ^line with the rpsL Janus cassette [21]. This required the introduction of a mutated *rpsL *gene to obtain a R6 transformed cell resistant to streptomycin (see Materials and methods). The integration of the PBP1a Janus cassette (Additional file 3) into an R6^2x2b-M2 ^streptomycin-resistant recipient (R6^SmR, 2x2b-M2^) was selected under kanamycin pressure and yielded streptomycin-sensitive and kanamycin-resistant R6^2x2b-M2, 1a::janus ^transformants. The replacement of the Janus cassette from R6^2x2b-M2, 1a::janus ^was performed in a second round of transformation with a *pbp1a *fragment amplified from R6M2 flanked by 3 kb of upstream and downstream regions (Additional files 3 and 4). The selection under streptomycin pressure yielded the streptomycin-resistant and kanamycin-sensitive R6^SmR, 2x2b1a-M2 ^transformants that acquired the *pbp1a *allele of R6M2. However, although the targeted sequencing of *pbp1a *in R6^SmR, 2x2b1a-M2 ^confirmed the acquisition of the R6M2 nonsense mutation at position 411 of the protein, this strain had penicillin resistance levels identical to those of the R6^2x2b-M2 ^line (MIC 0.125 μg/ml; Table 4). We tested further the role of PBP1a in resistance by introducing a wild-type copy of the gene in the penicillin-resistant mutant R6M1. The co-transformation of a *pbp1a *fragment amplified from *S. pneumoniae *R6 WT with a *rpsL *fragment conferring streptomycin resistance into R6M1 yielded the R6M1^SmR,1a-wt ^transformant that harbored a WT *pbp1a *allele. Interestingly, the R6M1^SmR, 1a-wt ^transformant became two times more sensitive to penicillin (PG MIC 1.0 μg/ml) than its parent (Table 4).

### The inactivation of spr1178 confers tolerance to antibiotic-induced oxidants

In addition to the mutations in *pbp *genes, the WGS highlighted three genes that were mutated in both the R6M1 and R6M2 mutants (spr1178, spr1254 and spr1888) but only spr1178 was also mutated in 1974M1 and 1974M2. Although an increased expression of the PstB ABC transporter subunit (spr1254) had previously been associated with penicillin resistance [17], its inactivation by insertional duplication mutagenesis in *S. pneumoniae *R6 WT did not translate into increased penicillin tolerance (Table 4). Similarly, the independent inactivation of spr1178 or spr1888 in a WT background had no effect on the level of penicillin susceptibility (Table 4). Given that the nonsense mutation in spr1178 had been acquired in every penicillin-resistant mutant analyzed, we tested whether this recurrence required a background of altered PBPs in order to confer resistance. Again, neither the independent transformation of the mutated versions of spr1178 and spr1254 into R6^SmR, 2x2b1a-M2 ^and R6^2x2b-M2 ^recipients nor the simultaneous transformation of the spr1178 and spr1254 mutations into an R6^2x2b-M2 ^recipient altered the penicillin susceptibility of the transformants (Table 4).

**Figure 1:**
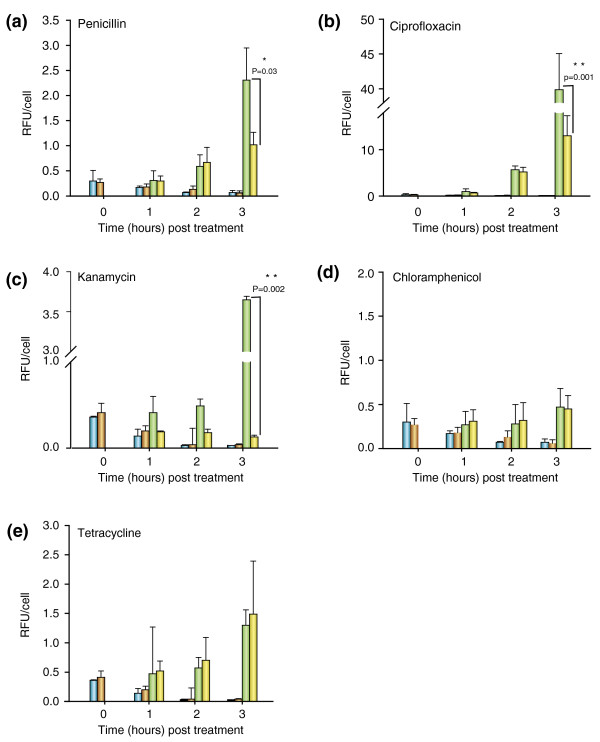
**Nonsense mutation in spr1178 and reduced accumulation of reactive oxygen species induced by bactericidal antibiotics**. Drug-induced reactive oxygen species accumulation in *S. pneumoniae*. The DCF-DA fluorescence signals of the *S. pneumoniae *R6^SmR, 2x2b1a-M2 ^(green bars) and R6^SmR, 2x2b1a, spr1178-M2 ^(yellow bars) transformants following exposure to **(a) **0.1 μg/ml penicillin, **(b) **4.0 μg/ml ciprofloxacin, **(c) **400.0 μg/ml kanamycin, **(d) **6.0 μg/ml chloramphenicol, and **(e) **0.25 μg/ml tetracycline were measured prior to antibiotic exposure (time zero) and 1 hour, 2 hours, and 3 hours following addition of the antibiotics. The DCF-DA fluorescence signals of the *S. pneumoniae *R6^SmR, 2x2b1a-M2 ^(blue bars) and R6^SmR, 2x2b1a, spr1178-M2 ^(orange bars) transformants untreated cultures measured at each time point are indicated as control. Results are the average of at least three independent experiments. RFU, relative fluorescence units.

Spr1178 encodes a protein of 192 amino acids with one predicted transmembrane domain that was categorized as part of the DUF3347 family of functionally uncharacterized proteins by Pfam analysis. However, a BLAST analysis for spr1178 homologues in different *Streptococcus *species revealed several proteins annotated as Fe^2+^/Pb^2+ ^permeases with at least 70% identity. Indeed, spr1178 had 86.9% and 86.7% similarity with a putative iron permease of the FTR1 family [22] from *Streptococcus gordonii *[23] and *Streptococcus mitis *ATCC6249, respectively, and 94% identity with a putative high-affinity Fe^2+^/Pb^2+ ^permease from *S. pneumoniae *670-6B, *S. pneumoniae *CDC3059-06 and *S. pneumoniae *P1031. Intriguingly, the bactericidal activity of antibiotics like β-lactams has recently been linked to the iron-dependent accumulation of reactive oxygen species (ROS) [24]. Since spr1178 has a predicted iron permease function, we sought to determine whether the acquisition of the nonsense mutation in spr1178 could translate into decreased accumulation of ROS following exposure to penicillin. Using the dichlorofluorescein diacetate (DCF-DA) dye, whose fluorescence intensity is proportional to the levels of ROS, we showed that sub-inhibitory concentrations of penicillin induced a greater time-dependent increase in ROS accumulation in a *S. pneumoniae *R6^SmR, 2x2b1a-M2 ^background than in cells in which the spr1178 nonsense mutation was introduced (Figure 1a). Similarly, penicillin induced a greater accumulation of ROS in R6 WT in comparison to a R6 WT strain in which spr1178 was disrupted (data not shown). We next tested whether this was a more general feature of bactericidal antibiotics and we found that ciprofloxacin (Figure 1b) and kanamycin (Figure 1c) also induced significantly more ROS in cells harboring an unaltered spr1178 gene. The time-dependent accumulation of ROS was a specific feature of bactericidal antibiotics, since the bacteriostatic antibiotics chloramphenicol (Figure 1d) and tetracycline (Figure 1e), respectively, failed to induce ROS even in the presence of a WT version of spr1178 and only induced a slight accumulation of ROS that was not correlated to the functional status of spr1178.

**Figure 2:**
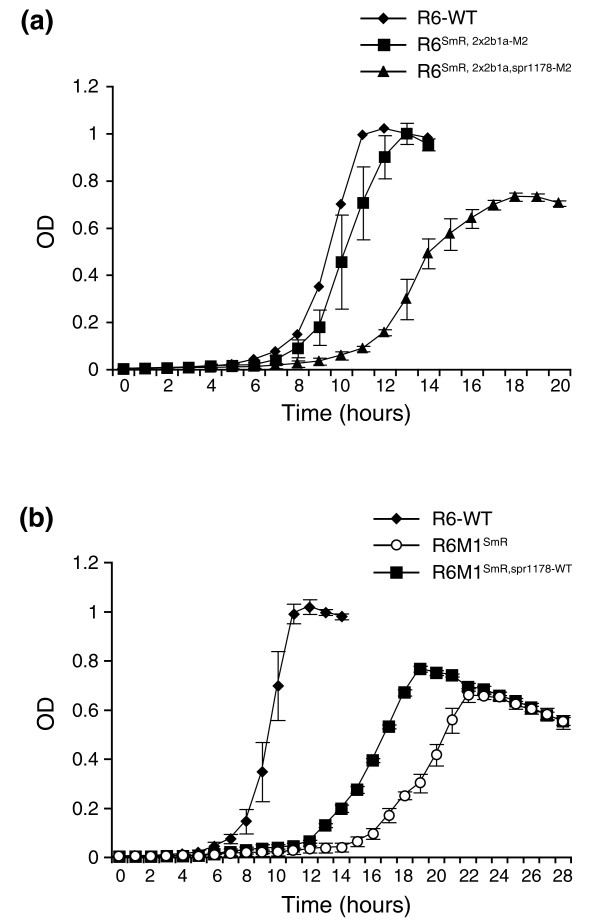
**Nonsense mutation in spr1178 and growth defect in penicillin-resistant *S. pneumoniae***. **(a) **The growth kinetics of *S. pneumoniae *R6 wild-type, *S. pneumoniae *R6^SmR, 2x2b1a-M2 ^and *S. pneumoniae *R6^SmR, 2x2b1a, spr1178-M2 ^was followed by measuring the optical density of the cultures every hour for a 20-hour period. **(b) **The reversion of the spr1178 nonsense mutation to a wild-type sequence decreased the growth defect of the R6M1 mutant. Results are the average of at least three independent measurements.

Growth kinetics revealed that penicillin resistance conferred a fitness cost to R6M1 (Figure 2b) and R6M2 (data not shown). The acquisition of PBP2x, 2b and 1a mutations was not associated with this growth defect, as the growth of the R6^SmR, 2x2b1a-M2 ^transformant was not altered compared to *S. pneumoniae *R6 WT (Figure 2a). In contrast, the introduction of a nonsense mutation in spr1178 conferred a fitness cost, as the growth of the R6^SmR, 2x2b1a, spr1178-M2 ^transformant was altered compared to its R6^SmR, 2x2b1a-M2 ^parent or to *S. pneumonaie *R6 WT (Figure 2a). Similarly, the introduction of a WT spr1178 allele restored the fitness of R6M1, although the growth defect could not be completely reverted (Figure 2b).

## Discussion

Whole genome sequencing of sensitive and resistant organisms is a powerful tool for understanding the biology of resistance mechanisms [18,19,25-27]. We sequenced two independent mutants selected for penicillin resistance *in vitro *to concentrate on recurrent mutations, a strategy proven to be useful [19]. Resistance to β-lactams in *S. pneumoniae *clinical isolates was shown to be a complex process involving the acquisition of PBP variants of low antibiotic affinity by intra- and interspecies gene transfer events from related streptococcal species [4,5]. Most PBP mutations directly involved in resistance were shown to alter the polarity and charge distribution around the catalytic cleft of the proteins. Indeed, the G552E substitution located in the vicinity of the active site of PBP2x [28] is a major determinant of β-lactam resistance [29-31] by inducing a decreased acylation efficiency to the protein [31]. Interestingly, several PBP2x mutations have been selected in our R6 and 1974 penicillin-resistant mutants, with every resistant strain except for R6M2 having acquired the Q552E substitution (Table 3). Other PBP2x mutations that have been specifically selected in at least one of our mutants include the A369V substitution in R6M2 that was previously reported to be one of the six PBP2x mutations responsible for the β-lactam resistance of *S. pneumoniae *clinical isolates [32] and the G601V substitution observed in 1974M1 that was shown to indirectly affect the active site of PBP2x by introducing a bulkier side chain involved in topological alterations of the catalytic cleft [8]. The F388L substitution selected in 1974M1 was shown to be one of the three substitutions responsible for cefotaxime resistance in a *S. pneumoniae *clinical isolate [8]. The F388L substitution is in the core of a hydrophobic niche close to the catalytic serine, along with the adjacent S389L change frequently observed in resistant isolates [29], and could be involved in conformational alterations of the catalytic cleft. Finally, other PBP2x mutations identified in our penicillin-resistant strains probably have more indirect roles in resistance, like the R384G and R426C substitutions selected in both R6 mutants. These substitutions have also been found in a previously described series of laboratory-derived cefotaxime-resistant mutants [33].

Reconstruction of resistance by the stepwise introduction of PBP mutations into a R6 penicillin susceptible background revealed an ordered appearance of mutations first in *pbp2x*, then in *pbp2b *and finally in *pbp1a*. Although the R426C and V518I substitutions in PBP2x only appeared at the third and fifth level of R6M2 selection (Additional file 2), transformation experiments failed at introducing these PBP2x substitutions. The R426C substitution was previously suggested to act as a compensatory mutation that requires a specific genetic background in order to be effective [8], which could provide a plausible explanation to our failure to transfer this mutation into the R6^2x-M2 ^transformant. PBP1a variants have been previously shown to confer high-level penicillin resistance only in the presence of low affinity PBP2x [34,35] and/or PBP2b [35]. In our study, however, the introduction of the PBP1a nonsense mutation from R6M2 into the R6^2x2b-M2 ^line failed to increase the level of resistance to penicillin. It also appeared that a specific PBP-unrelated genetic background is required for PBP1a to participate in resistance as the reversion of its mutation in R6M1 resulted in a twofold decrease in resistance (Table 4).

In previously characterized laboratory-derived penicillin and cefotaxime-resistant mutants [36], PBP variants associated with resistance occurred late during the selection process [37,38], suggesting that the initial increase in resistance during the first steps of selection involves non-PBP mutations. Similarly, the transfer of R6M2 PBP mutations to a penicillin-susceptible strain did not allow it to reach the resistance level of the parent mutant R6M2. Together, this implies that other mutations are probably involved in resistance. The analysis for recurrent mutations in our panel of resistant strains pinpointed a nonsense mutation in the putative iron permease spr1178 that occurred early during the selection process, before any PBP mutations could be selected (with the exception of the Q281P substitution in R6M2) (Additional file 2). It has recently been argued that bactericidal antibiotics, regardless of their primary targets, kill bacteria by inducing alterations in iron homeostasis, ultimately leading to the accumulation of hydroxyl radicals through the Fenton reaction [24]. Signaling events implicating the envelope stress-response and redox-responsive two-component systems were also found to be key players in triggering hydroxyl radical formation [39]. Although iron-sulfur clusters were initially implicated as the source of iron [24,40], the inactivation of TonB in *Escherichia coli *revealed that exogenous iron can also be implicated in the hydroxyurea-induced accumulation of ROS [41]. In this study, we have shown that three classes of bactericidal drugs, penicillin, ciprofloxacin and kanamycin, stimulate a greater production of ROS in the presence of a functional version of spr1178. Bacteriostatic antibiotics like tetracycline and chloramphenicol failed to induce ROS irrespective of the functional status of spr1178. Even though the transformation of the spr1178 nonsense mutation under a background of mutated PBPs did not reveal a direct role in resistance to penicillin, its early inactivation could have provided increased protection against the accumulation of ROS during the selection of resistance by potentially decreasing the availability of free iron. Moreover, the R6M2 mutant further harbors a nonsense mutation in another putative iron uptake system (spr0934; Table 2), which could potentiate the protective effect conferred by the spr1178 inactivation. The analysis of a panel of five penicillin non-susceptible clinical isolates failed to show similar nonsense mutations in spr1178 (data not shown), but this might in part be explained by the obvious growth defect associated with the acquisition of this mutation (Figure 2). It is salient to point out that the exposure to sublethal concentrations of bactericidal antibiotics was shown to induce a decreased expression of iron uptake systems in *Pseudomonas aeruginosa *[42] and *S. pneumoniae *[37], so similar gene expression alterations could also potentially occur in clinical isolates to prevent the accumulation of ROS during the early steps of resistance selection, instead of more drastic events like nonsense mutation as observed in isolates selected *in vitro*.

Our comparative genomic approach revealed that the selection for penicillin resistance in *S. pneumoniae *frequently involves the acquisition of a nonsense mutation in a putative iron transport system that increases the tolerance to antibiotic-induced accumulation of ROS. This tolerance should lead to an increased survival that putatively allows the selection of more important resistance determinants, such as the sequential accumulation of point mutations in PBPs.

## Conclusions

This study indicates that, for *in vitro *isolates, mutations in PBPs are not sufficient to achieve high level resistance to penicillin. Our study also reveals that penicillin kills cells by producing ROS, possibly through the Fenton reaction since less ROS are produced in resistant mutants in which a putative iron transporter is mutated. The whole genome sequencing data further revealed other mutations that were acquired by at least one mutant and we propose that some of these, or a combination of mutations, could be associated with penicillin resistance along with mutations in PBPs.

## Materials and methods

### Bacterial strains and culture conditions

All strains used in this study are listed in Table 4. Pneumoccoci were grown as previously described [19]. Clones of the *S. pneumoniae *R6 laboratory strain and the clinical isolate *S. pneumoniae *CCRI-1974 [19] were used for the laboratory-induced selection of penicillin resistance. The selection of resistance was performed on Zybalski plates containing concentration gradients of PG as described previously for other drugs [43]. For subculturing, colonies were picked in the area of highest antibiotic concentrations and streaked onto agar plates containing either the same concentration of antibiotic or a gradient of increased antibiotic concentrations. The MIC of the resistant cells isolated from the plates with the highest concentrations of antibiotic was determined to confirm the resistance phenotype. Five selection cycles were required to obtain the highly resistant M1 and M2 mutants for each strain.

### Antibiotic susceptibility

Antibiotic susceptibilities were determined with E-test strips (AB bioMérieux, Stockholm, Sweden) on Müller-Hinton agar plates supplemented with 5% sheep blood using the manufacturer's instructions. The MICs were further confirmed by the microdilution method according to the Clinical Laboratory Standards Institute (CLSI) guidelines.

### Whole genome sequencing

Genomic DNAs were prepared from mid-log phase *S. pneumoniae *cultures using the Wizard Genomic DNA Purification kit (Promega, Madison, WI, USA) according to the manufacturer's instructions. The genome of the R6M1 mutant was sequenced by using the NimbleGen WGS approach [44]. Briefly, DNA from the R6M1 mutant and its progenitor were differentially labeled with fluorescent markers and were co-hybridized on DNA tiling microarrays. Regions hybridizing differently were sequenced by a second round of sequencing hybridization arrays. The sequencing and analysis were performed by NimbleGen [20]. The genome of the R6M2 mutant was sequenced using the 454 Life Sciences (Roche, Branford, FL, USA) GS-FLX system. The genome sequencing, assemblies, and comparative analysis were performed at the McGill University Genome Quebec Innovation Center. The sequence of R6M2 is available at NCBI under the accession number PRJNA73471. Mutations, deduced from either array hybridizations or massively parallel sequencing, were confirmed by PCR amplification and Sanger sequencing. A circular map of the genome of R6M1 and R6M2 showing the identified mutations is provided (Additional file 1).

### DNA constructs

The genetic constructs used in this study are described in Additional file 5. Gene inactivation was done by insertional duplication mutagenesis using the nonreplicative pFF3 and pFF6 plasmids. The pFF3 plasmid is a pGEMT easy (Promega) derivative into which an Eam1105I restriction site was introduced in the multiple cloning site and the ampicillin resistance marker was replaced by the chloramphenicol resistance marker of pEVP3 [45] (a kind gift of D Morrison, University of Illinois at Chicago). The pFF6 plasmid is a derivative of pFF3 in which the chloramphenicol resistance marker was replaced by the kanamycin marker of pDL289 [46] (a kind gift of D Cvitkovitch, University of Toronto). Fragments of the genes to be inactivated were amplified from genomic DNA of *S. pneumoniae *R6 (Additional file 4) and cloned into the multiple cloning sites of pFF3 or pFF6. The Janus cassette (a kind gift from D Morrison, University of Illinois, Chicago) was also used for gene inactivation and gene replacement study in a streptomycin-resistant background in *S. pneumoniae *as described [21]. Janus is a 1.3-kb cassette with a kanamycin resistance marker and a counterselectable *rpsL *marker conferring streptomycin sensitivity. To generate a streptomycin-resistant background, *S. pneumoniae *strains were transformed with a *rpsL^+ ^*PCR fragment that was amplified from chromosomal DNA of the streptomycin-resistant strain CP1296 and the selection was done on agar supplemented with 150 μg/ml of streptomycin.

### Genetic transformation

The penicillin-resistance phenotype was reconstructed by transforming the penicillin-susceptible *S. pneumoniae *R6 with PCR fragments amplified from the penicillin-resistant R6M1 or R6M2 mutants (Additional file 4). Selection was done on plates containing appropriate concentrations of penicillin. Competent cells were obtained by the dilution of an overnight *S. pneumoniae *culture 1:100 in C+Y medium, pH 6.8 [47]. The diluted cultures were grown up to the onset of exponential phase before being concentrated ten times and frozen in C+Y, pH 6.8, 15% glycerol. For transformation, competent cells were thawed on ice, diluted ten times with C+Y medium, pH 7.8, and complemented with 2 μg/ml of competence stimulating peptide 1 (csp-1) before being incubated for 15 minutes at 35°C under a 5% CO_2 _atmosphere. DNA was added to a final concentration of 2 μg/ml and the cultures were incubated for 1 hour at 30°C. Finally, the cultures were switched to 35°C under a 5% CO_2 _atmosphere for 1 hour before being plated on CAT medium containing the appropriate concentration of antibiotic. The plates were incubated for 48 hours at 35°C under a 5% CO_2 _atmosphere and the resistant colonies were picked for further studies.

### Detection of reactive oxygen species

The intracellular ROS accumulation was measured using the DCF-DA dye (Invitrogen, Grand Island, NY, USA) whose fluorescence is proportional to the level of ROS [48]. In a typical experiment, cells were grown to the onset of exponential phase (OD_600 _0.12) before penicillin, ciprofloxacin, kanamycin, chloramphenicol or tetracycline were added at a final concentration of 0.1, 4.0, 400, 6.0 and 0.25 μg/ml, respectively. One milliliter aliquots were collected at baseline (prior to the addition of antibiotic) and at 1, 2 and 3 hours following the addition of antibiotic. The aliquots were washed once and resuspended in 500 μl of 1× PBS (pH 7.2) containing 5 μM DCF-DA and incubated at 37°C in the dark for 30 minutes. The labeled cells were washed once and resuspended in 500 μl of 1× PBS. The fluorescence signal of a 200 μl aliquot was analyzed using a Victor fluorometer (Perkin-Elmer, Waltham, MA, USA) at 485 nm excitation and 535 nm emission wavelengths. Results are expressed as relative fluorescence units (RFU) and were normalized according to the number of live cells at each time point. A minimum of three independent experiments have been performed for each antibiotic.

## Abbreviations

DCF-DA: dichlorofluorescein diacetate; MIC: minimum inhibitory concentration; PBP: penicillin-binding protein; PBS: phosphate-buffered saline; PCR: polymerase chain reaction; PG: penicillin G; ROS: reactive oxygen species; SNP: single-nucleotide polymorphism; WGS: whole genome sequencing; WT: wild-type.

## Competing interests

The authors declare that they have no competing interests.

## Authors' contributions

FF, DL and MO designed the study. FF performed the experiments, analyzed the data and drafted the manuscript; PL and DL revised the manuscript and provided critical comments. All authors approved the final version of the manuscript.

## Supplementary Material

Additional file 1 **Circular maps of the genome of R6M1 and R6M2**. Click here for file

Additional file 2 **Chronological appearance of PBP mutations according to the levels of penicillin resistance in R6M2**. Click here for file

Additional file 3 **PBP1a-targeting Janus cassette**. Click here for file

Additional file 4 **Oligonucleotides used in this study**. Click here for file

Additional file 5 **Plasmids used in this study**. Click here for file
