# Supplementary material for: Whole genome sequencing of penicillin-resistant Streptococcus pneumoniae reveals mutations in penicillin-binding proteins and in a putative iron permease
Source: Genome Biol. 2011 Nov 22;12(11):R115. doi: 10.1186/gb-2011-12-11-r115 (PMC3334601; doi:10.1186/gb-2011-12-11-r115)
Supplement: Additional file 1 — Circular maps of the genome of R6M1 and R6M2. [file gb-2011-12-11-r115-S1.DOC]

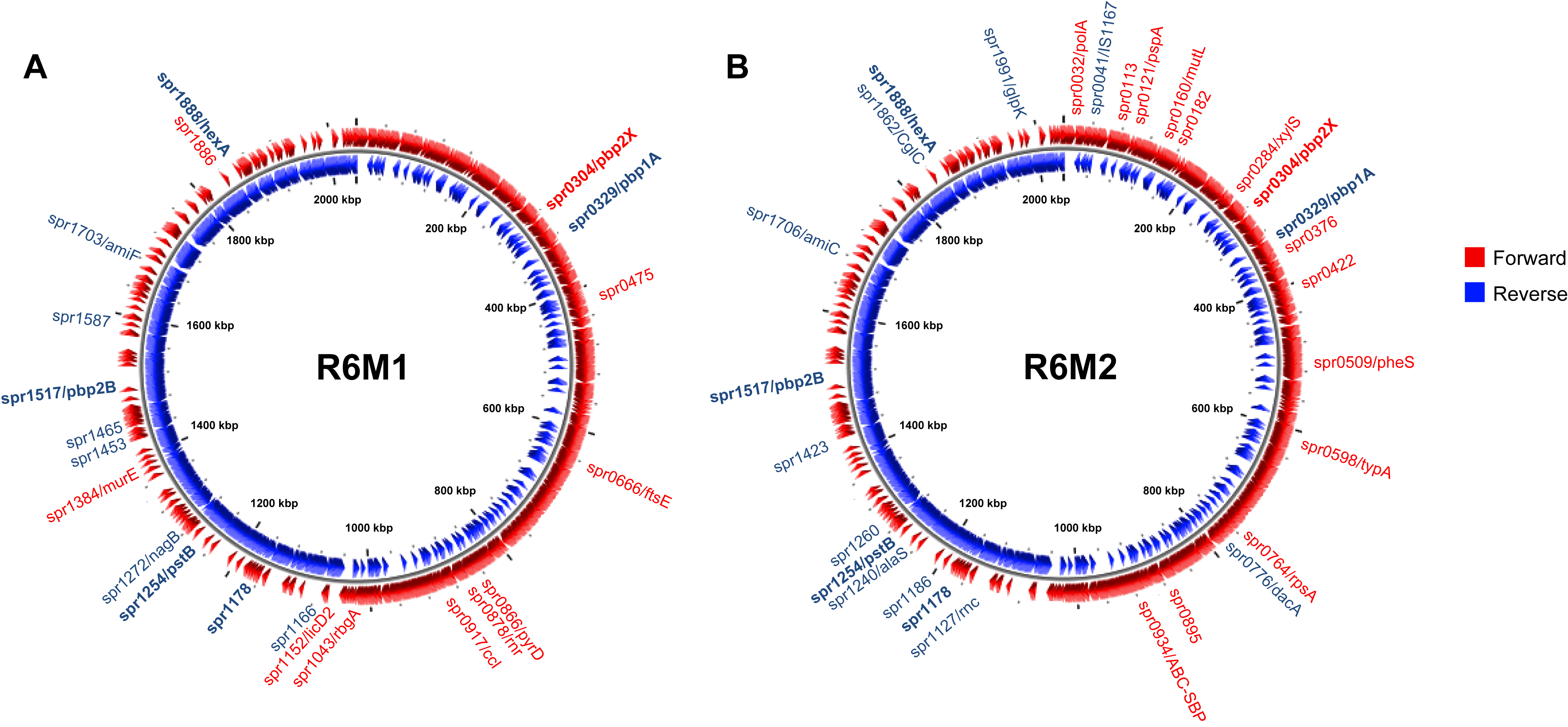


**Additional file 1. Circular maps of the genome of R6M1 and R6M2.**

Circular maps (CG view) of the R6M1 (A) and R6M2 (B) genomes in which the genes containing non-synonymous mutations are shown. The genes located on the forward strand are indicated in red and those located on the reverse strand are shown in blue. Recurrent mutations in both mutants are shown in bold. *polA*, DNA polymerase A; *IS1167*, transposase; *pspA*, surface protein precursor; *mutL*, DNA mismatch repair protein; *xylS*, alpha xylosidase; *pbp*, penicillin binding protein; *pheS*, phenylalanyl-tRNA synthetase; *typA*, tyrosin phosphorylated protein A; *ftsE*, cell division ATP binding protein; *rpsA*, 30S ribosomal protein S1; *dacA*, D-alanyl-D-alanine carboxypeptidase; *pyrD*, dihydrooratate dehydrogenase; *rnr*, exoribonuclease R; *ccl*, citrulline cluster-linked gene; *ABC-SBP*, ATP binding cassette-substrate binding protein; *rbgA*, ribosomal biogenesis GTPase; *rnc*, ribonuclease III; *licD2*, phosphorylcholine transferase ; *alaS*, alanyl-tRNA synthetase; *pstB*, phosphate transporter ATP-binding protein; *nagB*, N-acetylglucosamine 6-phosphate isomerase; *murE*, UDP-N-acetylmuramoyl-L-alanyl-D-glutamyl-L-lysine ligase; *ami*, N-acetylmuramoyl-L-alanine amidase; *cglC*, competence protein; *hexA*, hexoaminidase ; *glpK*, glycerol kinase. The maps have been generated with the CG view software (http://www.simgene.com/CGView).
