# Supplementary material for: Whole genome sequencing of penicillin-resistant Streptococcus pneumoniae reveals mutations in penicillin-binding proteins and in a putative iron permease
Source: Genome Biol. 2011 Nov 22;12(11):R115. doi: 10.1186/gb-2011-12-11-r115 (PMC3334601; doi:10.1186/gb-2011-12-11-r115)
Supplement: Additional file 2 — Chronological appearance of PBP mutations according to the levels of penicillin resistance in R6M2. [file gb-2011-12-11-r115-S2.DOC]

**Additional file 2**. **Chronological appearance of PBPs mutations according to the levels of penicillin resistance in R6M2**.

| Interval stage | PBP | | | | | | | | |  |
| --- | --- | --- | --- | --- | --- | --- | --- | --- | --- | --- |
| of M2 | PBP2x | | | | | PBP2b | | | PBP1a | Spr1178 |
| Level 1  MIC;0.06 | A842C  *Q281P* | NO | NO | NO | NO | NO | NO | NO | NO | C82T  *Q28** |
| Level 2  MIC;0.125 | A842C  *Q281P* | NO | NO | NO | NO | NO | NO | A1351G  *T451A* | NO | C82T  *Q28** |
| Level 3  MIC;0.25 | A842C  *Q281P* | NO | A1150G  *R384G* | C1276T  R426C | NO | NO | NO | A1351G  *T451A* | NO | C82T  *Q28** |
| Level 4  MIC;0.5 | A842C  *Q281P* | C1106T  *A369V* | A1150G  *R384G* | C1276T  R426C | NO | NO | NO | A1351G  *T451A* | NO | C82T  *Q28** |
| Level 5  MIC;1.0 | A842C  *Q281P* | C1106T  *A369V* | A1150G  *R384G* | C1276T  R426C | G1552A  *V518I* | C1184T  *A395V* | NO | A1351G  *T451A* | NO | C82T  *Q28** |
| Level 6  MIC;2.0 | A842C  *Q281P* | C1106T  *A369V* | A1150G  *R384G* | C1276T  R426C | G1552A  *V518I* | C1184T  *A395V* | G1303A  *G435S* | A1351G  *T451A* | G1233A  W411* | C82T  *Q28** |

Mutations are shown as nucleotide changes in the first line and the corresponding amino acid changes shown in italic on the second line. Asterix (***) indicates nonsense mutations. NO indicates no mutations. MICs are in µg/ml.
