# Supplementary material for: Whole genome sequencing of penicillin-resistant Streptococcus pneumoniae reveals mutations in penicillin-binding proteins and in a putative iron permease
Source: Genome Biol. 2011 Nov 22;12(11):R115. doi: 10.1186/gb-2011-12-11-r115 (PMC3334601; doi:10.1186/gb-2011-12-11-r115)
Supplement: Additional file 3 — PBP1a-targeting Janus cassette. [file gb-2011-12-11-r115-S3.PDF]

Additional file 3. PBP1a-targeting Janus cassette.

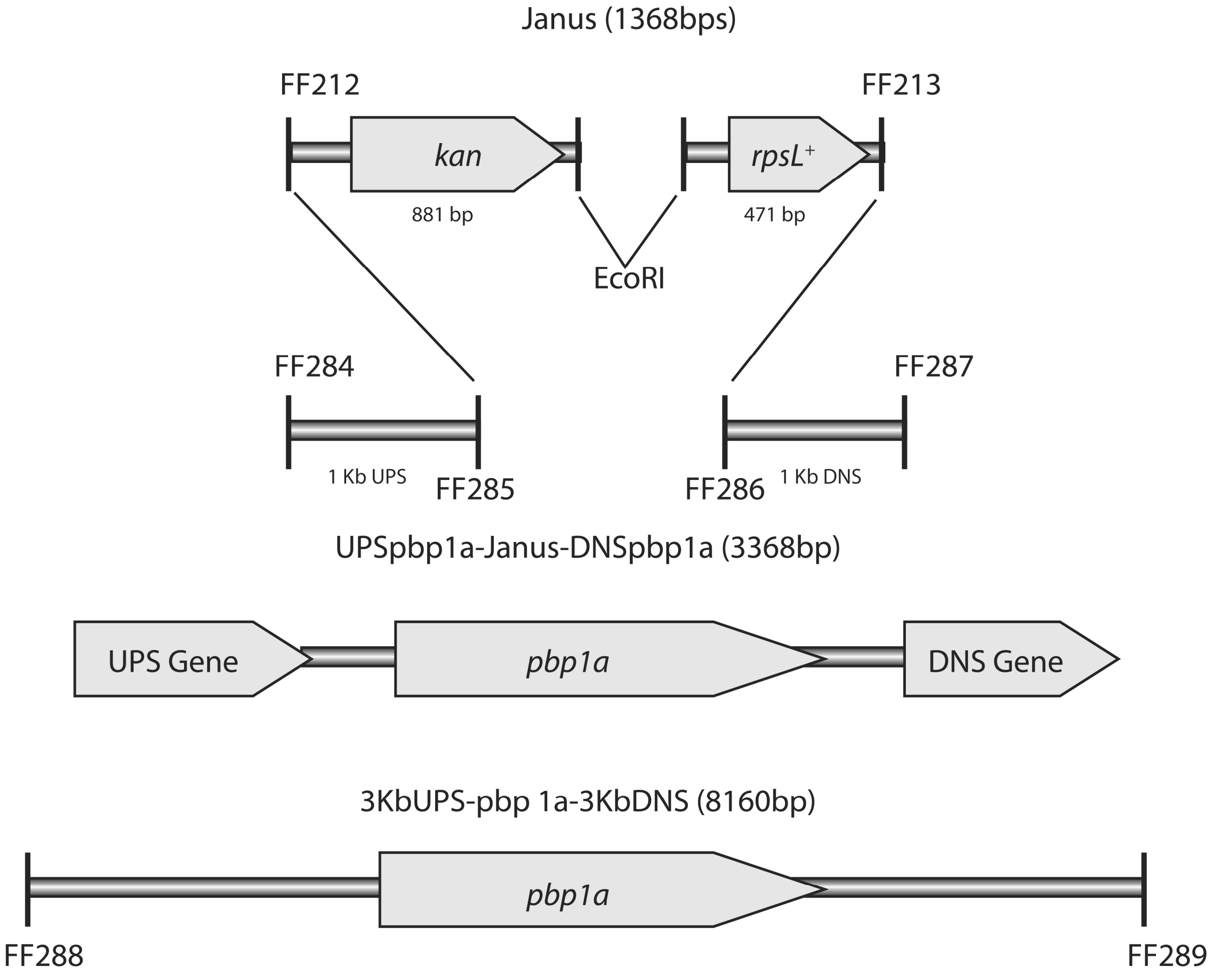

The *rpsL* and *kan*, modules of the Janus cassette are shown. Primers used to amplify two targeting fragments are indicated at the termini of those PCR products. Top, PCR fragment used to construct $R6^{2x2b\text{-}M2,\ 1a::Janus}$ transformant. Middle, *pbp1a* chromosomal region. Bottom, a 8,160-bp fragment containing *pbp1a* flanked with 3Kb upstream (UPS) and downstream (DNS) amplified fragments from chromosomal DNA of R6M2 with primers FF288 and FF289, This 8160 bp fragment was used for reintroduction of *pbp1a* of R6M2 to replace the Janus Cassette.
