# Supplementary material for: Whole genome sequencing of penicillin-resistant Streptococcus pneumoniae reveals mutations in penicillin-binding proteins and in a putative iron permease
Source: Genome Biol. 2011 Nov 22;12(11):R115. doi: 10.1186/gb-2011-12-11-r115 (PMC3334601; doi:10.1186/gb-2011-12-11-r115)
Supplement: Additional file 4 — Oligonucleotides used in this study. [file gb-2011-12-11-r115-S4.DOC]

**Additional file 4**. **Oligonucleotides used in this study.**

| Primer | Targeted Locus | Sequence (5’ to 3’) |
| --- | --- | --- |
|  |  |  |
| For pbp sequencing and PCR transformation | | |
| FF152 | pbp2b-F | CTAATTCATTGGATGGTATTT |
| FF153 | pbp2b-R | ATGAGACTGATTTGTATCAGA |
| FF154 | pbp2x-F | ATGAAGTGGACAAAAAGAGTA |
| FF155 | pbp2x-R | TTAGTCTCCTAAAGTTAATGT |
| FF156 | pbp1a-F | TTATGGTTGTGCTGGTTGAGG |
| FF157 | pbp1a-R | ATGAACAAACCAACGATTCTG |
|  |  |  |
| For gene replacement using Janus cassette | | |
| FF284 | 1KbUP- pbp1a -F | GTAGCAGATGACTTTGCAAGTCTTACAGCT |
| FF285 | UP- pbp1a -JANUS-KM-R | ATTTCCTCTGGAATAGGCATAGACATTTATCATCCAGATTTTT |
| FF286 | DN- pbp1a -JANUS-SM-F | AAAAGCATAAGGAAAGGGGCCCTAGCTTGTTTTACCACCTAATAA |
| FF287 | 1Kb- pbp1a -R | CCCCTTGTGTTCATAGCGAGGATAAGCA |
| FF212 | JANUS-F | TCTATGCCTATTCCAGAGGAAATGGAT |
| FF213 | JANUS-R | CTAGGGCCCCTTTCCTTATGCTTTTGGAC |
| FF288 | 3KbUP- pbp1a -F | TCAACAGTGCCATCACCCGC |
| FF289 | 3KbDN- pbp1a -R | ATAAGGCGGATTGGAAATGATTACTCC |
|  |  |  |
| For point mutation transformation of Non-PBPs | | |
| FF235 | 2.5KbUP-spr1178-F | AAAGTTGTTAAACCTCCGCAAAAACC |
| FF236 | 2.5KbDN-spr1178-R | AACACCGATATCACCAGCAGGTA |
| FF237 | 2.5KbUP-spr1254-F | TTATCAATTTGCCAAGGTGTCCC |
| FF238 | 2.5KbDN-spr1254-R | GTCAATGTCCAAGGGGGAGG |
|  |  |  |
| For gene inactivation | | |
| FF158 | Spr1254-F-KO | CTCAAACATGGTCTCTTCTAG |
| FF159 | Spr1254-R-KO | GTCAACCGTCCAGAAATCAAC |
| FF207 | Spr1178-F-KO | TGTTTCAACACGTCCATAAT |
| FF208 | Spr1178-R-KO | CTAGTTTTAGCTTTGTCATTTTT |

Underlined sequences correspond to complement of Kanamycin (KM) and Streptomycin (SM) parts of the Janus cassette. UP, upstream; DN, downstream; F, forward; R, reverse.
