# Supplementary material for: Whole genome sequencing of penicillin-resistant Streptococcus pneumoniae reveals mutations in penicillin-binding proteins and in a putative iron permease
Source: Genome Biol. 2011 Nov 22;12(11):R115. doi: 10.1186/gb-2011-12-11-r115 (PMC3334601; doi:10.1186/gb-2011-12-11-r115)
Supplement: Additional file 5 — Plasmids used in this study. [file gb-2011-12-11-r115-S5.DOC]

**Additional file 5. Plasmids used in this study.**

| Plasmid or cassette | Description | Source |
| --- | --- | --- |
| Janus Cassette | A 1.3-kb cassette containing a KM resistance marker and conferring SM sensitivity for gene replacement through negative selection in *S. pneumoniae*. | (Sung et al. 2001) |
| pFF3 | *S. pneumoniae* non-replicative vector that contains a CM resistance marker | Generated in our lab  (unpublished data) |
| pFF6 | pFF3 in which the CM resistance marker was replaced with a KM resistance marker. | Generated in our lab  (unpublished data) |
| pFF3-1178 KO | pFF3 carrying an internal segment of spr1178 | This study |
| pFF6-1254 KO | pFF6 carrying an internal segment of spr1254 | This study |

KM, Kanamycin; SM, Streptomycin; CM, Chloramphenicol.
